# Body Composition as a Modulator of Bone Health Changes in Patients with Inflammatory Bowel Disease

**DOI:** 10.3390/life12020272

**Published:** 2022-02-12

**Authors:** Iulia Soare, Anca Sirbu, Miruna Popa, Sorina Martin, Cristian George Tieranu, Bogdan Mateescu, Mircea Diculescu, Carmen Barbu, Simona Fica

**Affiliations:** 1 Faculty of Medicine, Carol Davila University of Medicine and Pharmacy, Dionisie Lupu Street, no. 37, Sector 2, 020021 Bucharest, Romania; iulia.soare@drd.umfcd.ro (I.S.); anca.sirbu@umfcd.ro (A.S.); miruna.popa@drd.umfcd.ro (M.P.); sorina.martin@umfcd.ro (S.M.); cristian.tieranu@umfcd.ro (C.G.T.); radu.mateescu@umfcd.ro (B.M.); mihai.diculescu@umfcd.ro (M.D.); simona.fica@umfcd.ro (S.F.); 2 Elias University Hospital, Marasti Street, no. 17, Sector 1, 011461 Bucharest, Romania; 3 Colentina Hospital, Stefan cel Mare Bd, no. 19-21, Sector 2, 072202 Bucharest, Romania; 4 Fundeni Institute, Calea Bucuresti, no. 258, 022328 Bucharest, Romania

**Keywords:** bone health, IBD, TBS, fat mas, ASMI, VAT

## Abstract

Background: Bone impairment of multifactorial etiology is a common feature in inflammatory bowel disease (IBD). Body composition parameters, which might be selectively modified in these patients, are important determinants of bone strength. Our aim was to investigate the relationship between components of body composition and bone parameters in IBD patients. Methods: This is a cross-sectional, retrospective study including 80 IBD patients (43 women, 37 men). Lumbar spine (LS), femoral neck (FN) and whole body DXA scans were performed to analyze regional bone mineral density (BMD), as well as body composition, including appendicular skeletal muscle mass index (ASMI), total and visceral fat mass (VAT). Trabecular bone score (TBS) was assessed using iNsight Software. Results: Twenty (25%) IBD patients had inadequate LS-BMD z scores (<=−2DS). Lean mass (LM) was a significant determinant of LS-BMD, after adjusting for age, gender, BMI and fat mass (*p* < 0.01), while fat mass% remained associated with FN-BMD (*p* < 0.01). TBS correlated positively with BMI (*r* = 0.24, *p* < 0.05), LS-BMD (*r* = 0.56, *p* < 0.001), ASMI (*r* = 0.34, *p* < 0.001) and negatively with VAT/total fat% (*r* = −0.27, *p* < 0.05). Multivariate analysis showed that ASMI, LS-BMD (positively) and VAT/total fat% (negatively) were independently associated with TBS. Conclusions: In IBD patients, skeletal muscle mass and fat percentage and distribution are important factors associated with bone health.

## 1. Introduction

Inflammatory bowel disease (IBD), which comprises Crohn’s disease (CD) and ulcerative colitis (UC), represents a chronic inflammatory condition associated with a significant impairment in all aspects of an individual’s life [1]. Although prone to malabsorption and malnutrition due to multiple factors (chronic inflammatory disease, increased risk for surgeries and low oral intake secondary to anorexia as well s diarrhea), about 15–40% of IBD patients are obese [2] and a significant number have abnormalities in body composition [3]. Bone impairment is common in these patients, with a prevalence of approximately 12–70%, even in newly diagnosed patients [4], and fracture risk is increased with 30–40% compared to the general population [5].

The impact of body mass index (BMI) on bone mineral density (BMD) is well known, and for many years, increased body weight was regarded as a protective factor against bone impairment [6]. However, BMI might underestimate the risk for low BMD as body weight is composed of lean mass (LM), fat mass (FM), fluids and bone mass. LM has been shown to positively impact bone quality, both in women and men [7]. Fat mass might also be an important component, with a significant impact on BMD values due to mechanical loading and hormonal mechanisms [8], including leptin [9] and insulin action [10]. However, data are contradictory when we look at adiposity compartments and their distinct metabolic profiles, with some studies showing a detrimental impact of adiposity on bone [11]. Furthermore, the fracture risk is increased in overweight individuals, especially in the ankle, humerus and foot [12]. 

Altered body composition is common in IBD patients [13]. Decreased LM is frequent in these patients and has been associated with osteopenia [14]. Visceral adipose tissue (VAT), the “creeping fat” [15], has been considered to be highly relevant in IBD patients, especially in CD, as it is an important secreting site of mesenteric adipocytes, releasing proinflammatory cytokines, such as TNFα. Chronic inflammation has been associated with increased risk for osteoporotic fractures in IBD patients [16]. Whether assessed through imaging techniques (CT, MRI and DXA), or estimated by means of waist circumference, VAT has been widely reported as a negative predictor for bone quality [17,18,19,20,21,22]. Moreover, it has been studied as a potential marker for relapse and activity of the disease, which itself could aggravate bone loss [23]. 

Dual-energy X-ray absorptiometry (DXA) is generally used in patients with IBD for the measurement of BMD, has a major role in estimating fracture risk and it is also a very important tool for the evaluation of body composition [3]. However, it is well known that bone strength and quality depend not only on density, but also on skeletal characteristics that are not measured by DXA, including bone size and geometry, microarchitecture of trabecular and cortical compartments, microdamages, bone turnover and composition of the mineralized protein matrix [24]. Trabecular bone score (TBS) is a novel method, derived from DXA image of the lumbar spine, which indirectly assess trabecular microstructure. A large number of studies have demonstrated the utility of TBS in evaluating bone microarchitecture and, along with clinical factors and BMD, in estimating fracture risk through FRAX analysis [25,26]. TBS has shown its utility, especially in secondary osteoporosis and, in particular conditions, such as glucocorticoid induced osteoporosis or diabetes mellitus, it might outperform DXA [22].

Overall, a modified body composition, with decreased lean mass and increased fat mass percentage has been generally shown to have a detrimental impact on bone health. However, data regarding the impact of these alterations in IBD patients are scarce. The aim of our study was to evaluate the association between DXA-derived parameters of body composition and variables of bone mineralization (BMD) and bone quality (TBS) in a group of IBD patients.

## 2. Materials and Methods

This study has a cross-sectional design, comprising 80 IBD patients (48 with CD and 32 with UC) registered in three gastroenterology tertiary centers and referred for endocrinological evaluation to Elias Hospital Endocrinology Department in Bucharest, Romania, where the study was conducted. All the patients were adults diagnosed according to European Crohn and Colitis Organization guidelines [27]. The exclusion criteria included other causes of secondary osteoporosis and pregnancy. The study protocol was conducted in accordance with the Declaration of Helsinki and was approved by the Ethics committees of the Carol Davila University of Medicine and Pharmacy and Elias Hospital (Bucharest, Romania). All patients provided their informed consent.

Using DEXA Prodigy®, GE Healthcare no. 212018, Madison, WI, United States, values of the bone mineral density (BMD) (g/cm^2^) of the lumbar spine and femoral neck were calculated. For lumbar BMD, we excluded affected vertebrae, such as those with fractures of degenerative changes. Trabecular bone score (TBS) measurements were performed using TBS iNsight version 3.0.2.0 (Medimaps Group, Plan-les-Ouates, Switzerland) and calculated for the same region as the LS BMD assessment. 

Weight and height were determined for all patients using a digital scale (in kg) and stadiometer (in cm), without wearing shoes and with light clothes in the morning. We also performed whole body DXA examination for all patients, which provided values for LM (kg), fat mass (in kg and percentage) and fat distribution. For the characterization of LM, we used ASMI, calculated as the sum of the arms’ and legs’ muscle mass divided by square height, in accordance with the European Working Group on Sarcopenia in Older Patients and the International Working Group on Sarcopenia guidelines [28,29]. Fat mass index (FMI) was calculated by dividing fat mass by square height. Visceral adipose tissue (VAT) mass and volume were also estimated via DXA. Myopenia was defined as Z score <−1 DS [28] with population-based and age- and gender-matched data values from the National Health and Nutrition Examination Survey (NHANES) [30].

Obesity was defined using WHO definition, as a BMI >= 30 kg/m^2^ [31], while a body fat percentage above 33 in women and 25 in men was considered abnormal [32].

Statistical analysis was performed using SPSS version 21.0 (SPSS). Descriptive data were presented as mean ± SD or median (interquartile range) based on the variable type. Pearson and Spearman correlation analysis were used for parametric and nonparametric variables, respectively. Non-normally distributed variables were logarithmically transformed before being included in linear regression analysis, which was used to determine the influence of different parameters on TBS. Independent t test (for normally distributed variables) and the Mann–Whitney U test (for nonparametric variables) were used to compare men and women groups. Linear regression was used to identify independent factors for DXA parameters. *p* value of < 0.05 was considered statistically significant.

## 3. Results

### 3.1. Patients’ General Characteristics

Of our 80 patients, with a mean age of approximately 43 years, 47 (58.7%) were diagnosed with CD and 33 (41.2%) had UC. Table 1 summarizes the main characteristics of these IBD patients, according to gender. There were no significant differences between men and women regarding age, type of disease or the majority of bone densitometry parameters (LS and hip BMD z scores, TBS). However, despite a relatively similar BMI, women had higher fat mass% and lower fat free mass, LM and ASMI (*p* < 0.001) compared to men. Looking at the visceral adiposity, women also had lower VAT mass and volume, as well as a significantly decreased VAT/total fat mass ratio (*p* < 0.05). Twenty (25%) IBD patients had inadequate lumbar BMD z scores (<=−2DS). No difference regarding bone impairment prevalence was observed between CD and UC patients, or between men and women.

### 3.2. Relationship between Body Composition Parameters and BMD 

#### 3.2.1. Lumbar Spine BMD

**Figure 1:**
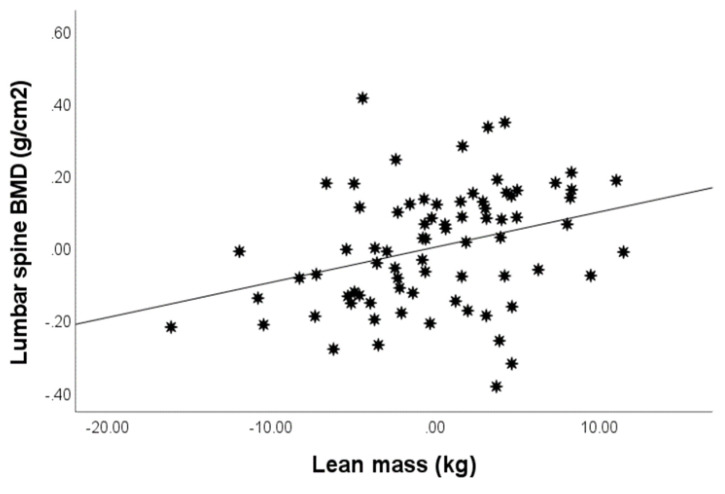
Positive correlation between lean mass and lumbar spine BMD, after adjusting for gender, age, BMI and fat mass.

Lumbar spine BMD positively correlated with BMI (*r* = 0.260, *p* < 0.05), fat mass (*r* = 0.208, *p* < 0.05) and LM (*r* = 0.389, *p* < 0.01). Notably, LS-BMD correlation with LM remained highly significant even after adjusting for age, gender, BMI and fat mass (*p* < 0.01, Figure 1).

#### 3.2.2. Femoral Neck BMD

**Figure 2:**
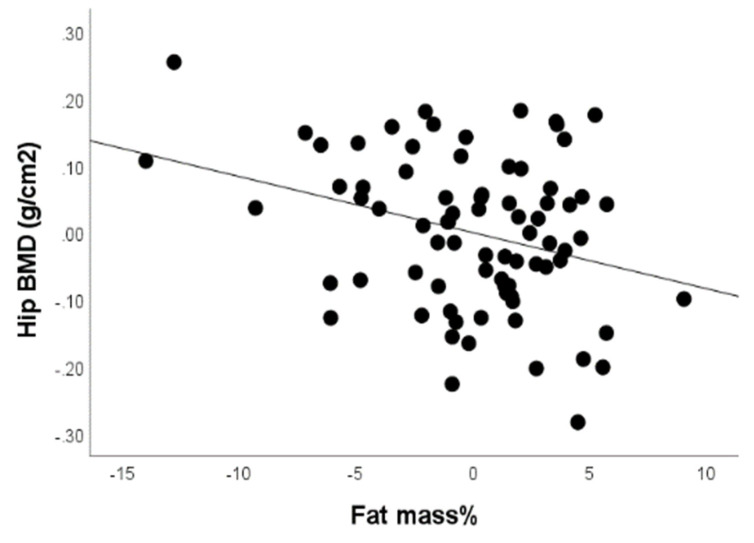
Negative correlation between fat mass% and hip BMD after adjusting for BMI, age and lean mass.

In a similar manner, we tried to identify factors associated with total hip BMD values. Femoral neck BMD was lower in women (Table 1) and positively correlated with BMI (*r* = 0.201, *p* < 0.05), fat free mass (*r* = 0.433, *p* < 0.01), LM (*r* = 0.435, *p* < 0.01). Conversely, there was a negative relationship between hip BMD and fat mass% (*r* = −0.196, *p* < 0.05), as well as age (*r* = −0.224, *p* < 0.05). To assess which of the variables significantly associated with hip BMD in the preliminary analysis are in fact independent contributors to its variability, we performed multiple regression linear analysis. Factors independently associated with hip BMD were BMI (*p* < 0.01), age (*p* < 0.01) and fat mass% (*p* < 0.01), in a model that also included LM and gender (*r*^2^ for model = 0.35, *p* < 0.001). The relationship between fat mass% and hip BMD is depicted in Figure 2.

### 3.3. Determinants of Bone Quality Measured by Trabecular Bone Score (TBS)

TBS values were not correlated with patients’ age (*r* =−0.2, *p* = 0.08), gender (Table 1) or the type of IBD (mean TBS = 1.38 ± 0.11 in Crohn patients, 1.38 ± 0.1 in UC patients, *p* = 0.9). As expected, there was a very strong positive correlation between TBS and lumbar BMD (*r* = 0.561, *p* < 0.001, and a positive correlation between TBS and BMI (*r* = 0.245, *p* < 0.05). In univariate analysis, after adjusting for BMD and BMI, TBS positively correlated with ASMI (*r* = 0.344, *p* < 0.01) and negatively with fat mass% (*r* = −0.228, *p* < 0.05), VAT (−0.285, *p* < 0.05) and VAT/total fat % (*r* = −0.274, *p* < 0.05). 

Considering the important differences in body composition between men and women (as seen in Table 1), we decided to perform separate regression models for each gender. In men, the best model, which explained over 60% of TBS variance, identified spine BMD and especially VAT/total fat% as independent predictors of TBS values (Table 2). On the other hand, in women, the optimal model established spine BMD and ASMI as determinants independently associated with TBS. Finally, when considering all IBD patients, both VAT/total fat% and ASMI qualified as parameters independently influencing bone quality evaluated through TBS.

## 4. Discussion

In this study we evaluated the association between parameters of body composition (fat mass, visceral adiposity and lean mass) and DXA measured estimates of bone quantity and quality, such as lumbar and femoral neck BMD or TBS, in a group of patients with inflammatory bowel disease. We showed that, in this pathology group, lean mass is positively and independently associated with LS-BMD as well as TBS. Conversely, body adiposity seems to negatively relate to FN-BMD, while visceral adiposity is a parameter also negatively correlated with TBS.

IBD patients are known to have alterations in body composition and these disturbances might have negative effects on IBD-related outcomes, response to therapy or quality of life [3]. Obesity prevalence is rising in these patients, with reported rates between 10–40% [2]. Only 17% of our IBD patients could be classified as obese, according to the WHO definition, but 27.8% had increased body adiposity (body fat percentage z score above 1), with a significantly higher prevalence in men. Quite similar data were reported by Sigurdsson et al. in a study including 94 young adults with childhood onset IBD, in which 25% of patients had increased body adiposity [33]. 

The impact of these alteration on BMD is controversial. Almost 20 years ago, Blum demonstrated an inverse association between body fat percent and BMD, after adjusting for BMI, in premenopausal women [34]. More recently, Gandham et al showed that obese people (defined as increased body fat percentage) have an increased risk of incident fractures, whereas those who are obese according to BMI have reduced likelihood of fractures [35]. On the other hand, a study including almost 3800 Chinese aged >65 showed that fat mass percentage remained positively associated with lumbar and hip bone density even after adjusting for fat free mass [36]. In addition to this unclear association in the general population, data regarding the impact of adiposity on bone mass in IBD patients are scarce. In our study, fat mass percentage was negatively correlated with FN-BMD, especially after adjusting for BMI and LM, but this was not observed in association with LS-BMD. We identified just one other study marginally investigating this topic, in Sigurdsson’s study, they showed a tendency toward a negative correlation between fat % Z-score and FN- BMD z score, but without statistical significance [33].

As research and means of evaluation are evolving, attention has shifted from bone quantity to bone quality, on the one hand, and on the other hand, from BMI and fat mass to the differential function and impact of adipose compartments. TBS is an indirect measurement of bone microarchitecture, reflecting trabecular number and connectivity [37] and has been shown to predict fracture risk, independently of BMD [38,39]. 

Our data show positive correlation between BMI and TBS. Since TBS derives from LS BMD assessment, this could be a possible explanation. However, data from the literature are conflicting, with some authors describing a negative relationship between the two parameters [40,41]. Depending on DXA manufacture, BMI negatively correlated with TBS in Hologic measurement, but not in GE lunar [42], this difference being a result of the manner in which the software attenuates abdominal tissue mass. The original algorithm yielded lower TBS values in men than women, owing to the greater amount of abdominal fat mass [43]. However, when the algorithm was updated, a positive correlation between TBS and BMI was described [44].

Looking into determinants of TBS, the proportion of VAT and ASMI emerged as positive and negative predictors, respectively. Interestingly, there were obvious gender-specific differences, with VAT/fat% exhibiting a stronger correlation to TBS in men, while ASMI exhibited a stronger correlation in women. Comparative analysis between men and women showed a significant higher fat mass and lower ASMI in women than men. A previous study described no statistical differences between fat mass in men vs. women with IBD [14]; however, a possible explanation could reside in their inclusion of younger patients. Additionally, although not statistically significant, in our cohort, women were older than men. 

In one of the earlier studies regarding this topic, Gilsanz and colleagues employed CT scans for the assessment of both VAT and bone quality in 100 healthy young women and revealed that VAT had a negative impact on bone quality measures (in contrast to subcutaneous fat, which was a positive prediction factor) [17]. Similar results were reported by Kim et al. in a study using DXA to evaluate the relationship between body composition and TBS in 1474 Korean postmenopausal women [20] and by Lv et al. in a study on healthy Chinese men [19]. Conversely, in the Framingham Offspring cohort, VAT showed no correlation to bone quality measures after adjusting for BMI [45].

Several mechanisms have been proposed as explanations for the complex bone-VAT crosstalk, ending up in detrimental skeletal effects. Firstly, android fat, especially VAT, is associated with an overexpression of proinflammatory cytokines, such as TNFα, IL-1 and IL-6, involved in bone resorption and osteoclastogenesis, but also exerting potential negative influence on osteoblast differentiation [46,47,48]. Moreover, it appears TNF-α and IL-1β can upregulate osteoblast and adipocyte expression of 11β-hydroxysteroid dehydrogenase type 1, the enzyme converting cortisone to cortisol, enhancing the negative impact of cortisol on bone balance [48,49]. This comes as a supplementary burden in IBD, itself a condition of chronic inflammation. 

Adipokines, such as leptin and adiponectin, have been classically associated with positive effects of adipose tissue of bone homeostasis, such as induction of osteoblast proliferation and inhibition of osteoclastogenesis [50] Both of these molecules have a lower expression in VAT compared to SAT [51,52,53] and similar findings were reported for aromatase [54], which might explain the lack of a positive effect. However, it seems adiponectin can also negatively impact the bone, by means of increasing RANK-L activity and decreasing osteoprotegerin [55]. The relationship is further complicated in IBD since, for example, leptin has been shown to increase during exacerbations of UC [56], while higher levels of adiponectin correlate to a greater degree of inflammation in CD [57].

Finally, we also need to consider the fact that VAT can exert a negative impact on bone quality through the promotion of a more severe evolution of IBD. The hypertrophy of mesenteric adipose tissue in CD has long been recognized and is positively correlated with a more severe prognosis [58], while a higher VAT/SAT ratio seems to be correlated to a more aggressive phenotype [59,60]. 

In our study, ASMI showed positive correlations with both BMD and TBS. The apparent favorable impact of LM on LS BMD is consistent with previous results shown in both healthy individuals [61] and IBD patients [14]. In a Manitoba IBD cohort study, LM was the strongest determinant of BMD and its variation was associated with changes in bone density [62]. Sarcopenia is highly prevalent in IBD patients and can develop through a variety of mechanisms, including malnutrition, chronic inflammation, increased inflammatory status in adipose tissue, vitamin deficiency and imbalance of the muscle–gut axis [63]. Strategies to increase LM include, among other, physical exercise and this was recently shown to modulate the relationship between muscle and bone density in young people with IBD [64]

As for the correlation between ASMI and TBS, the former was an independent positive predictor in young, healthy persons, regardless of gender [65]. Moreover, a similar correlation was found in the elderly, even after adjusting for age, smoking, alcohol and BMI [66]. Recent research suggests that the influence of muscle on bone tissue goes beyond mechanics and is also based on the secretory profile of the muscle, with its various myokines rendering it an actual endocrine organ [67,68,69].

The strength of our study is that, to our knowledge, this is one of the few research projects investigating the association between body composition (fat mass percentage and distribution, total LM and relative appendicular mass indices) and bone quantity and quality, in patients with inflammatory bowel disease. However, our study has some limitations. Firstly, the number of patients included was relatively small. We are aware that other important factors, such as disease activity and duration or glucocorticoid exposure, are major parameters that could interfere with bone loss in IBD patients. Recently we have shown that the Harvey–Bradshaw index, a marker of disease activity, is independently associated with TBS values in CD patients, while exposure to high-dosage glucocorticoids has a deleterious effect on both BMD and TBS values [70]. However, body composition is affected by disease activity and glucocorticoid therapy and changes such as increased fat mass percentage and visceral adiposity as well as decreased LM and sarcopenia might modulate the negative impact of other risk factors. Another limitation of this study is the lack of a control group; however, considering the scarcity of studies regarding this topic on IBD patients, we believe that our data would improve the knowledge and contribute to a more profound understanding of the association between an unhealthy body composition and bone impairment in IBD patients.

## 5. Conclusions

In conclusion, we showed that body composition parameters are important determinants of bone mass quantity and quality in IBD patients. Fat mass percent and visceral adiposity seem to have a deleterious impact since they were negatively associated with BMD and TBS, respectively, while preserving LM has a beneficial effect. Since this was a small sample and transversal research, further prospective studies are needed to confirm these associations, to explore the mechanisms behind this cross-talk and to investigate whether body composition changes are indeed a link between factors associated with disease severity and impairment in bone quality.

## Figures and Tables

**Table 1:** Patients’ characteristics, according to gender.

Variable	Women (*n* = 43)	Men (*n* = 37)	Total (*n* = 80)	*p* (W vs. M)
Age (years)	47 (25)	38 (19)	44 (24)	0.291
Crohn’s disease (%)	55.8	62.2	58.7	0.653
BMI (kg/m^2^)	24.55 (6.03)	24.4 (7.32)	24.5 (7.5)	0.562
LS BMD (g/cm^2^)	1.04 ± 0.16	1.08 ± 0.2	1.06 ± 0.18	0.345
LS BMD z score (SD)	−0.75 ± 1.13	−1.07 ± 1.56	−0.91 ± 1.35	0.297
FN BMD (g/cm^2^)	0.85 ± 0.12	0.92 ± 0.13	0.89 ± 0.13	0.045
FN BMD z score (SD)	−0.71 ± 0.86	−0.83 ± 1.02	−0.77 ± 0.93	0.559
TBS	1.37 ± 0.1	1.39 ± 0.11	1.38 ± 0.11	0.282
Obesity (%)	16.3	18.4	17.3	0.771
Fat mass (kg)	23.68 (12)	21.9 (16)	22.5 (12)	0.653
Fat free mass (kg)	39.35 (9)	54.4 (13)	46.2 (15)	0.007
Fat mass (%)	39.29 ± 8.29	31.66 ± 8.53	35.76 ± 9.19	<0.001
Excessive fat mass (patients), (%)	16.7	40.5	27.8	0.005
FMI (kg/m^2^)	9.66 (5.35)	7.03 (4.24)	7.8 (5.2)	0.065
VAT mass (g)	605 (1162)	946 (1332)	714 (1370)	0.005
VAT/FAT (%)	2.49 (3.27)	3.63 (3.51)	3.14 (3.22)	<0.001
Lean mass (kg)	38.05 ± 5.69	51.69 ± 8.67	44.35 ± 9.91	<0.001
ASMI (kg/m^2^)	6.34± 0.9	7.5 ± 1.2	6.9 ± 1.2	<0.001
Myopenia (%)	55.3	27.9	41.2	0.003

Values are presented as mean ± SD or median (IQR), according to the type of variable and the normality of distribution. *p*-value presented statistically significant differences *p* < 0.05. BMI: body mass index; LS: BMD lumbar spine bone mineral density; FN: BMD femoral neck bone mineral density; TBS: trabecular bone score; FMI: fat mass index; VAT: visceral adipose tissue; ASMI: appendicular skeletal muscular index.

**Table 2:** Multiple regression analysis of TBS determinants.

		Men *r*^2^ = 0.602, *p* < 0.001		Women *r*^2^ = 0.422, *p* < 0.001		Total *r*^2^ = 0.470, *p* < 0.001	
Dependent Variable	Independent Variables	β	*p*	β	*p*	β	*p*
TBS	BMI (kg/m^2^)	0.302	0.563	−0.588	0.218	−0.441	0.198
	Lumbar BMD (g/cm^2^)	0.268	0.049	0.443	0.002	0.404	<0.001
	VAT/total fat %	−0.619	<0.001	−0.263	0.156	−0.270	0.008
	ASMI (kg/m^2^)	0.183	0.587	0.566	0.031	0.683	0.005
	Fat mass%	0.179	0.582	0.383	0.297	0.313	0.196

## Data Availability

The data presented in this study are available on request from the corresponding author.
